# Synthesis and Physiochemical Properties of Sulphated Tamarind (*Tamarindus indica* L.) Seed Polysaccharide

**DOI:** 10.3390/molecules29235510

**Published:** 2024-11-21

**Authors:** Sabrina Ziliani, Anna Alekseeva, Carlo Antonini, Emiliano Esposito, Fabio Neggiani, Marco Sansò, Marco Guerrini, Sabrina Bertini

**Affiliations:** 1 Department of Materials Science, University of Milano-Bicocca, 20125 Milan, Italy; s.ziliani3@campus.unimib.it (S.Z.); carlo.antonini@unimib.it (C.A.); 2 Istituto Farmaco Biologico Sperimentale, Via Carducci, 64/D, 56017 San Giuliano Terme, Italy; f.neggiani@ifbs.it (F.N.); m.sanso@ifbs.it (M.S.); 3 Institute of Chemical and Biochemical Research G. Ronzoni, Via G. Colombo 81, 20133 Milan, Italy; alekseeva@ronzoni.it (A.A.); esposito@ronzoni.it (E.E.); guerrini@ronzoni.it (M.G.)

**Keywords:** enzymatic hydrolysis, LC-MS, molecular weight, NMR, structural characterization, sulphated polysaccharide, tamarind seed polysaccharide, viscosity

## Abstract

Tamarind seed polysaccharide (TSP) is a neutral water-soluble galactoxyloglucan isolated from the seed kernel of *Tamarindus indica* with average molecular weight (Mw) 600–800 kDa. The high viscosity of TSP slows solubilisation, and the absence of charged substituent hinders the formation of electrostatic interactions with biomolecules. TSP was sulphated in a one-step process using dimethylformamide as a solvent, and sulphur trioxide-pyridine complex as a sulphating reagent. Studies of chemical structure, molecular weight distribution and viscosity were conducted to characterise the synthesised products. The sulphation degree was established by conductimetric titration; the sulphate group distribution was studied by NMR spectroscopy and liquid chromatography-mass spectrometry, and sulphated TSP oligomers were obtained by enzymatic degradation with cellulase and/or xyloglucanase. Sulphated products showed higher solubility than TSP, Mws in the range of 700–1000 kDa, a sulphation degree of two to four per subunit and pseudoplastic behaviour. A preliminary study of mucoadhesion revealed the unexpected interaction of S-TSP with mucin, providing a route by which sulphated TSP interactions with biomolecules may be influenced.

## 1. Introduction

**Figure 1:**
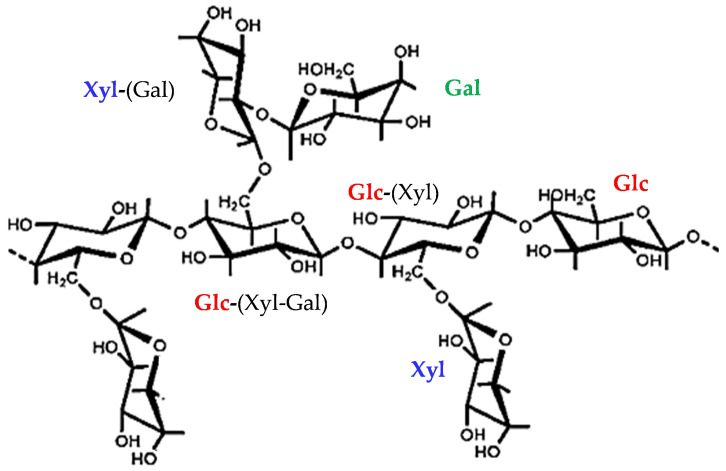
Chemical structure of tamarind seed polysaccharide (TSP) isolated from the seed kernel of *Tamarindus indica*. TSP is composed of a β-(1,4)-d-glucan backbone, with α-(1,6)-d-xylose branches, partially substituted with β-(1,2)-d-galactose. The coloured residues and abbreviations in bold correspond to the observed monosaccharide, while the linked monosaccharides are shown in parentheses. Glc—glucose; Xyl—xylose; Gal—galactose.

Tamarind seed polysaccharide (TSP) is a galactoxyloglucan isolated from the seed kernel of *Tamarindus indica*. It is a neutral and water-soluble polysaccharide composed of a β-(1,4)-d-glucan backbone with α-(1,6)-d-xylose branches that are partially substituted with β-(1,2)-d-galactose (Figure 1) [1]. It is applied as a stabiliser, thickener, gelling agent and binder in food and pharmaceutical industries due to its physical, chemical and biological properties, that include broad pH tolerance, biocompatibility, high thermal stability and low toxicity [2]. TSP can form viscous solutions when dissolved in water, and its “mucin-like” molecular structure determines its excellent mucoadhesive properties [3]. For this reason, it is applied in the ophthalmic field in the formulations of synthetic eye drops used for the treatment of the Dry Eye Disease (DED). In fact, its branched structure allows the adhesion to glycoprotein mucins of the ocular surfaces, and this interaction confers on TSP a higher residence time on the ocular surface [4].

Despite the above, there is room to further improve TSP properties and processing.

Owing to its high molecular weight and the absence of charged substituents, the solution containing the polysaccharide can become very viscous, and solubilisation of TSP requires a long time, and sometimes even heating [5]. For these reasons, formulations containing TSP at low concentrations are often prepared to facilitate the dissolution step, representing a limitation for some applications in the pharmaceutical field and beyond. TSP chemical modification by sulphated groups is desirable to extend processability and applicability. In particular, the sulphated modification can allow the formation of specific binding to proteins or receptors, significantly increasing the biological activity and the potential applications of the biopolymer [6]. These may include the use of sulphated TSP derivatives as mimics of glycosaminoglycans, natural sulphated polysaccharides which interact with a wide range of proteins involved in physiological and pathological process [7] including cell signalling and development, angiogenesis, axonal growth, tumour progression, metastasis and anti-coagulation [8]. Moreover, these sulphated polymers control blood clotting and play key roles in the attachment of pathogens, neuronal growth, cancer cell apoptosis and many other activities [9].

Since glycosaminoglycans are natural products from animals, their availability is limited, and so alternatives are sought. One possible source, glucosaminoglycans, sulphated polysaccharides from marine algae (carrageenan, ulvan, fucoidan) also possess important biological activity, as they have antitumor, antiviral, antioxidant, antimicrobial, anticoagulant and immune-inflammatory effects [10,11]. The increasing interest in these polysaccharides is explained by excellent source availability; however, their use remains a challenge due to different factors as the seasonality, the variability of macroalgae species and the extraction methods [12,13].

In contrast to the marine polysaccharides, TSP is a low-cost by-product of tamarind pulp industry [14]. It has a well-defined underlying structure [3] but retains considerable complexity [5]. Moreover, its high molecular weight, compared to those from marine algae [15], make TSP an attractive biopolymer for chemical modification to facilitate new applications.

The aim of this work was to determine the best conditions for TSP sulphation by tuning the extent of sulphation while maintaining high molecular weights.

The chemical structure, the molecular size and the degree of sulphation (DS) were characterised using a range of complementary physico-chemical techniques. Moreover, the stability toward enzymatic degradation by xyloglucanase, viscosity and mucoadhesive properties of S-TSP were evaluated.

## 2. Results

### 2.1. Sulphation Synthesis

TSP sulphation synthesis was performed in dimethylformamide, DMF, using sulphur pyridine complex (SO_3_xPy), modifying the sulphation methods of Nguyen [16] and Lang and Crescenzi [17]. In contrast with their published method, dry TSP was directly solubilised in DMF and was not precipitated in a gelatinous state before adding the reagent. Initially, the reaction was performed at room temperature (RT) and at 50 °C as reported by Lang and Crescenzi [17], at the same concentration of reagents and with a TSP/SO_3_xPy molar ratio of 1:1. The sample obtained at room temperature, S-TSP_1, showed a lower degree of sulphation than the product sulphated at 50 °C, S-TSP_1a (Table S1). However, S-TSP_1a showed lower absolute zeta potential and lower molecular weight, as reported in Table S1, due to depolymerisation of the product caused by the higher temperature. For this reason, the sulphation synthesis with higher concentration of reagent were performed at room temperature. TSP was solubilised at two different concentrations, 10 mg/mL and 5 mg/mL, to evaluate how this parameter influenced synthesis. As reported in Table 1, different TSP/SO_3_xPy molar ratios were tested to obtain S-TSP with different sulphation degrees.

#### 2.1.1. Conductimetric Titration

By conductimetric titration, the sulphation degree (DS) was determined, considering that 21 hydroxyl groups can be sulphated on the repeating unit of TSP (Figure 1). The DS values are reported in Table 1. By increasing the reagent, an increase in DS was observed, as expected. S-TSP_1 and S-TSP_2 show, respectively, DS of two and three and S-TSP_3 DS of four, meaning that a lower TSP concentration also leads to an increase in DS.

#### 2.1.2. Zeta Potential

Qualitative analysis of the sulphation reaction was performed measuring sample Zp, a parameter typically obtained by model-dependent transformation of the measured electrophoretic mobility used to estimate the surface charge of the polymers [18]. As expected, the Zp of neutral TSP is equal to 0 mV, while negative Zp values were determined for the sulphated TSP products (Table 1). Additionally, the absolute Zp value correlated with the degree of sulphation obtained by conductimetric titration: −43 mV for S-TSP_1, −47 mV for S-TSP_2 and −58 mV for S-TSP_3 (Table 1).

#### 2.1.3. FT-IR

**Figure 2:**
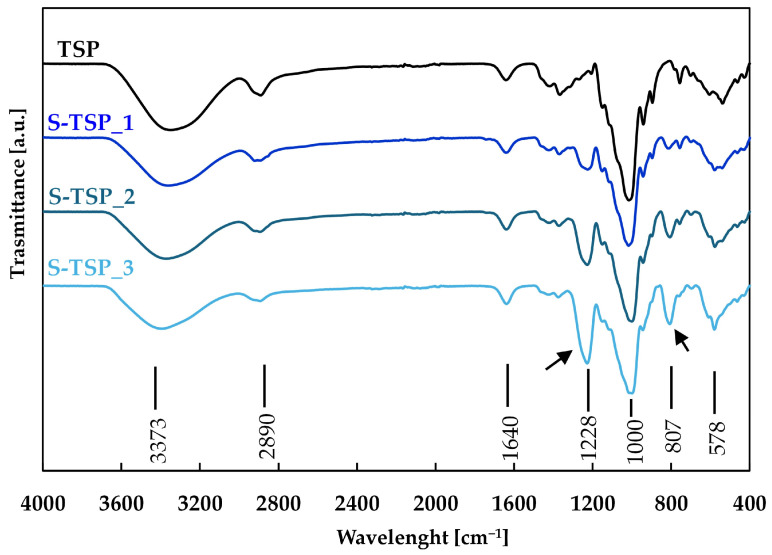
FT-IR spectra of TSP in black, S-TSP_1 in dark blue, S-TSP_2 in blue and S-TSP_3 in light blue. The new bands assigned to S=O and C–O–S stretching vibrations are signed with arrows.

TSP sulphation was also confirmed by FT-IR spectroscopy. The pristine TSP FT-IR spectrum (Figure 2) showed the typical profile of this polysaccharide [19]. The wide absorption peak at about 3347 cm^−1^ corresponded to the stretching vibration of hydroxyl groups (O–H) induced by inter- or intramolecular motion. The peak at 2890 cm^−1^ was due to C–H stretching and bending vibrations including CH, CH_2_ and CH_3_. Furthermore, peaks from 1638 cm^−1^ to 1372 cm^−1^ were also related to C–H bands [3]. The peak from 1150 to 897 cm^−1^ was attributed to C–O–C stretches [19].

In S-TSP compounds, the presence of the sulphated groups was confirmed by the formation of two new bands in the spectra at 807 cm^−1^ and 1227 cm^−1^, assigned to S=O and C–O–S stretching vibrations, respectively [17]. The intensities of these bands increased with increasing DS, from S-TSP_1 to S-TSP_3.

### 2.2. Chemical and Physical Properties

#### 2.2.1. Molecular Weight Distribution

**Figure 3:**
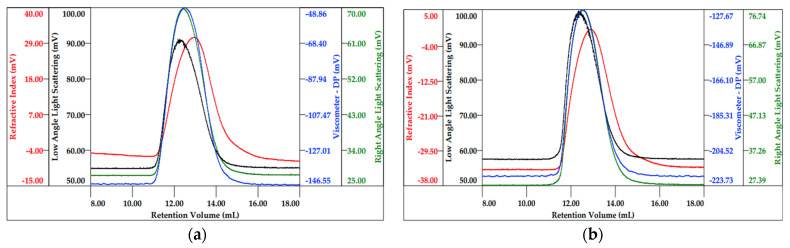
Chromatographic profile (red—refractive index; black—low laser light scattering; green—right angle light scattering; blue—viscometer) of pristine TSP (**a**) and S-TSP_1 (**b**).

HP-SEC-TDA with multi-detector systems (Right Angle and Low Angle Light Scattering, Refractive Index and Viscosimeter) was used to determine the molecular weight distribution, intrinsic viscosity, hydrodynamic radius and Mark–Houwink parameters of TSP and S-TSP products. After various preliminary tests, 0.3 M sodium acetate with 0.05% NaN_3_ (pH~8.1) was chosen as the mobile phase. The samples had an elution volume between 11 and 16 mL with a broad bell-shaped chromatographic peak, caused by a high polydispersity index (Figure 3). The chromatograms of TSP and S-TSP_1 are reported in Figure 3, while those of S-TSP_2 and S-TSP_3 are shown in the Supplementary Materials (Figure S1). Since no published *dn*/*dc* values are available for TSP and S-TSP, they were independently determined under the optimised chromatographic conditions: 0.139 mL/g and 0.125 mL/g were obtained for TSP and S-TSP, respectively. Mw (weight average molecular weight), Mn (number average molecular weight), polydispersity (expressed as Mw/Mn ratio), µ (intrinsic viscosity), Rh (hydrodynamic radius) and *a*, corresponding to the slope of the Mark–Houwink curve, are reported in Table 2. All the results refer to the mean values of duplicate injections.

The TSP Mw and Mn were 620 kDa and 405 kDa, respectively; S-TSPs showed higher molecular weight values compatible with the presence of sulphated groups on the polysaccharide chain. The molecular weight increase indicated that the reaction occurred without (or with negligible) depolymerisation, except for S-TSP_1a, which showed a decreasing of the molecular wight due to the increased temperature (Table S2). The Mw/Mn remained constant and similar to the starting TSP, indicating that there was no increase in polydispersity after derivatisation. The hydrodynamic radius (Rh) for TSP and S-TSP remain in the same range, expect for S-TSP_3, which showed a higher hydrodynamic radius of 41 nm due to higher molecular weight. The intrinsic viscosity, [η], of sulphated products is lower compared to TSP. This is because the introduction of sulphate groups along the chains leads to an increase in structural density, resulting in a decrease in viscosity [20]. Comparing the different S-TSP products, the [η] increases with the degree of sulphation, as the effect of increased molecular weight predominates over the increase in density. The Mark–Houwink parameter, *a*, is similar for all the samples and comparable to data reported in the literature [21]. The values of *a* are in the range of 0.7–0.9, indicating that, regardless of DS, the molecules remain in a random coil conformation. The weight recovery, determined by the refractive index area, is in a range between 80% and 92% for all the samples, and this value is compatible with the water absorption by dry samples.

#### 2.2.2. Rheological Properties

**Figure 4:**
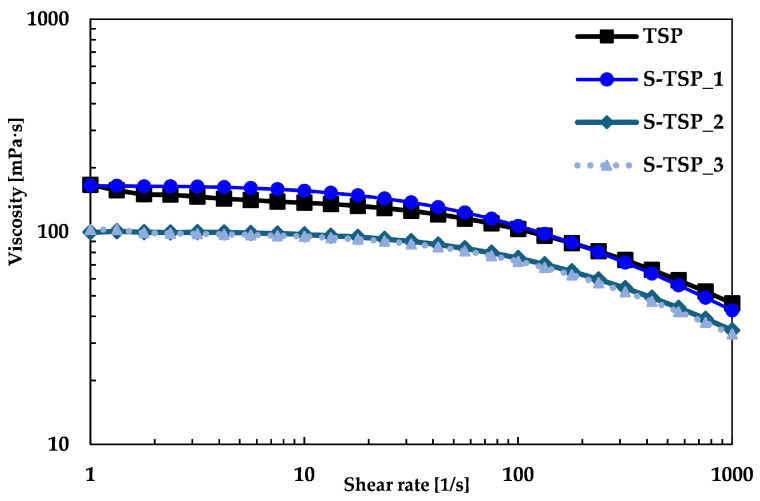
Viscosity curves of TSP in black, S-TSP_1 in dark blue, S-TSP_2 in blue and S-TSP_3 in light blue at 10 mg/mL at 20 °C.

The compound viscosity was also studied using a rheometer; Figure 4 shows that TSP and S-TSP behaved like pseudoplastic liquids, with viscosity decreasing for increasing shear rate. S-TSP_1 showed a very similar viscosity with TSP, starting at 165 mPa·s; in contrast, S-TSP_2 and S-TSP_3 had lower viscosity, starting at almost 100 mPa·s, probably due to the presence of the sulphated groups, which changes the conformation of the polysaccharide and decrease the viscosity [6].

Regarding the trend of viscosity, the more sulphated samples (S-TSP_2 and S-TSP_3) showed lower viscosity compared to TSP, but they were quite similar to each other, unlike the values determined by HP-SEC-TDA, for which the intrinsic viscosity increased from S-TSP_1 to S-TSP_3. It is important to emphasize that intrinsic viscosity, [η], is measured under infinite dilution conditions and is thus influenced by intramolecular rather than intermolecular interactions. In contrast, viscosity, which provides insights into the behaviour of a fluid under a variety of conditions, is not measured under infinite dilution, making intermolecular interactions significant. In this case, the negative charges of the sulphate groups are likely to play a crucial role in the viscous behaviour, counteracting the increase in molecular weight, which is not the predominant parameter.

### 2.3. Structural Properties of TSP, S-TSP and Their Enzymatically Hydrolysed Products

TSP and S-TSP products were characterised using monodimensional (1D) and bidimensional (2D) NMR spectroscopy. In the proton spectra (Figure S2), no significant chemical shift changes were observed following functionalisation. This is likely to be due to the overlap of broad signals caused by the high viscosity of the samples and the high molecular weight, which reduce the spectral resolution. Consequently, only the well-separated peaks were attributed to those of the anomeric region, specifically to xylose linked to galactose at 5.16 ppm, xylose linked only to glucose at 4.95 ppm, glucose and galactose at approximately 4.55 ppm and the hydrogen at position 2 of glucose at 3.40 ppm.

**Figure 5:**
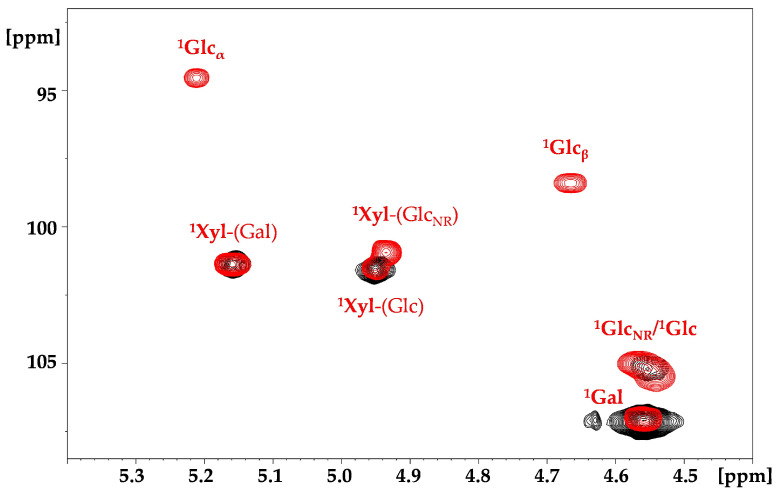
^1^H-^13^C HSQC superimposition of the anomeric region of TSP in black and hydrolysed TSP, with xyloglucanase in red. The superscript corresponds to the carbon number of the observed monosaccharide, which is in bold, while the monosaccharide linked is in the parentheses. NR—non-reducing end; Glc—glucose; Xyl—xylose; Gal—galactose.

2D ^1^H-^13^C heteronuclear single quantum correlation (HSQC) NMR allows for the resolution of overlapping signals present in ^1^H NMR and is widely used for characterisation of complex neutral [22] and charged [23] polysaccharides. The ^1^H-^13^C HSQC cross peaks of TSP, attributed in accordance with the literature data [24], are shown in Figure 5. In addition to the cross peaks related to glucose, galactose and xylose, signals from arabinose were also observed, likely due to contamination in plant seeds from arabinan or arabinoxylan, as reported in the literature [21,25].

In the HSQC NMR spectrum of S-TSP, the CH_2_ cross peaks at about 4.33–4.22/69.9 ppm, which are separated from the other backbone signals (blue circles in Figure S3), are consistent with sulphation of the primary alcohol of galactose and/or glucose [26].

As with the proton spectra, the resolution remains low because of the high molecular weight, which may prevent the detection of other signals related to sulphation. Moreover, as previously demonstrated with high molecular weight hyaluronic acid [27], two-dimensional experiments are not quantitative due to the different conformational mobility of the residues. Indeed, in the anomeric region of the TSP (Figure 5), galactose, which has greater conformational mobility compared to the other residues but is present in lower molar ratios (Glc:Gal = 3.1:1) relative to the theoretical unit [5], displays higher intensity than xylose and glucose. To address this, enzymatic degradation was performed to reduce the molecular weight of the polysaccharide while maintaining the substitutions, to allow a more effective NMR study. The lower molecular weight of the S-TPS products was expected to make LC-MS analyses feasible. Xyloglucanase and cellulase, enzymes with different specificity towards xyloglucans, were used: xyloglucanase hydrolyses the β-1,4 bond, primarily forming the repeating unit of TSP [21]; cellulase, expected to be less specific and less sensitive to branching, cleaves both the β-1,4 linkage between glucose units and 1,2-linked xylose and galactose [28]. After 24 h of depolymerisation of TSP, oligomers with Mw < 2 kDa similar to the theorical Mw of the repeating unit were obtained, regardless of the enzyme used. For S-TSP, the enzymes were less active due to the presence of sulphate groups. Consequently, a mixture of both enzymes was used, and the hydrolysis time was extended compared to that used for TSP.

#### 2.3.1. NMR Hydrolysed TSP

**Figure 6:**
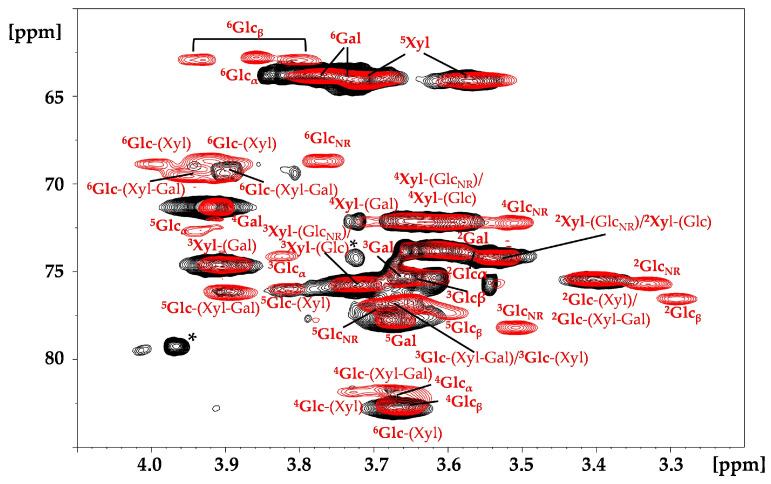
^1^H-^13^C HSQC superimposition of TSP in black and hydrolysed TSP with xyloglucanase in red. The superscript corresponds to the carbon number of the observed monosaccharide, which is in bold style, while the monosaccharide linked is in the parentheses. * signals assigned to the arabinose residue. Glc—glucose; Xyl—xylose; Gal—galactose.

HSQC spectra of hydrolysed TSP samples were recorded to verify the cleavage site of the enzymes. As shown in Figure 5, xyloglucanase hydrolysed only the linkage between the backbone glucose units. Indeed, in the anomeric region, the signals of the α/β reducing ends (REs) of the glucose were observed (Figure 5). In contrast, cellulase also cleaved the β-1,2 linkage between the xylose and the galactose, as indicated by the signals of the galactose monomeric unit (Figure S4). With both enzymes, a new signal at 4.93/100.9 ppm, attributed to the xylose linked to the non-reducing (NR) end glucose, was also observed (Figure 5). Owing to the short oligosaccharides, new signals in the hydrolysed TSP appeared to the non-hydrolysed pristine TSP, particularly in the region from 4.10 to 3.20 ppm. The complete attribution of the hydrolysed TSP obtained by xyloglucanase digestion is reported in Table 3 and in Figure 6.

A quantitative HSQC analysis of the sample treated with xyloglucanase was possible due to the reduced molecular weight of the product. The 1:1 ratio between galactose and xylose linked to galactose, along with a Glc:Xyl:Gal of 3.7:3:1.3, confirmed the proposed structure of the repeating unit (Supplementary Material in Figure S5).

#### 2.3.2. LC-MS of Enzymatically Hydrolysed TSP and S-TSP

**Figure 7:**
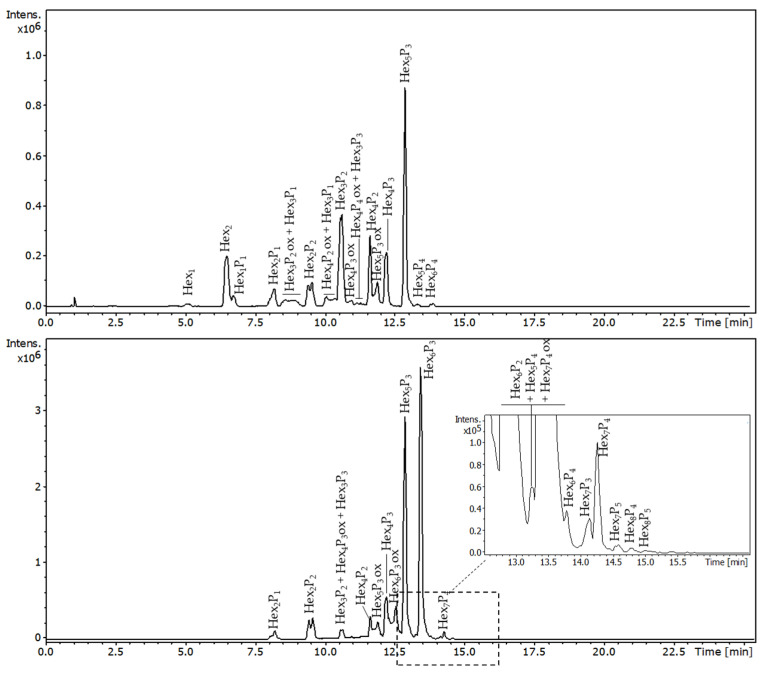
HILIC/ESI-QTOF-MS chromatograms of TSP hydrolysed by cellulase on the top and TSP hydrolysed by xyloglucanase on the bottom. Hex—hexose (glucose or galactose, 162 Da), P—pentose (xylose, 132 Da); the numbers in subscript indicate the number of hexoses and pentoses within the detected oligosaccharide: ox indicates the oxidized minor components (−2 Da), most likely related to the C4-oxidized oligomers, as previously reported by Sun et al. [29].

The cellulase and xyloglucanase generated digestion products of TSP were also analysed by hydrophilic interaction chromatography (HILIC) coupled with an ESI-QTOF mass spectrometer. The LC-MS profile of TSP hydrolysed by xyloglucanase (Figure 7) showed two major peaks attributed to oligomers, the first composed of five hexoses and three pentoses (Hex_5_P_3_, *m*/*z* 611.21 at 12.5 min) and the second composed of six hexoses and three pentoses (Hex_6_P_3_, *m*/*z* 692.24 at 13.5 min), both corresponding to the main repeating unit of the TSP (Figure 1), compatible with the values obtained by the integration of the NMR bidimensional spectra [21]. As expected, the less selective cellulase gave rise to shorter oligomers than xyloglucanase (Figure 7). Overall, the shorter oligomers are more abundant in the TSP treated with cellulase. The most intense peak corresponds to the oligomer composed by five hexoses and three pentoses (Hex_5_P_3_, *m*/*z* 611.21 at 12.5 min), as that previously obtained with xyloglucanase, while Hex_6_P_3_ was detected only in trace amounts. The observed differences between the products obtained by two enzymes are in accordance with the known higher specificity of xyloglucanase. It is most likely that the cellulase was able to cut the linkage between the xylose and the galactose, leading to the formation of Hex_5_P_3_. This hypothesis is in accord with the generation of monosaccharide galactose determined by NMR in cellulase-degraded TSP (discussed below).

Interestingly, both enzymes generate a short oligomer composed by two hexoses and two pentoses (Hex_2_P_2_, *m*/*z* 605.21 at 9.4 min, Figure 7). This oligomer can be unambiguously attributed to the structure containing two glucoses, both branched with xylose. Probably the enzymes hydrolysed two glucoses even if they were both linked with the xylose, with this fragment derived from longer oligomers with four or more xylose units near each other.

The disaccharide Hex_2_ that was detected only in the LC-MS analysis of cellulase-degraded TSP was attributed to the sucrose present in the cellulase as a stabiliser.

The LC-MS analyses also showed minor oligomers with a −2 Da mass shift compared to the regular TSP oligomers, suggesting possible oxidation of one of the hydroxy-groups to a C=O group. Such a side oxidation degradation of xyloglucans has been previously studied by MALDI-TOF [29]. The authors reported that the C=O may be generated at the NRE of the formed oligosaccharides at the level of enzymatic cleavage of the xyloglucan backbone.

**Figure 8:**
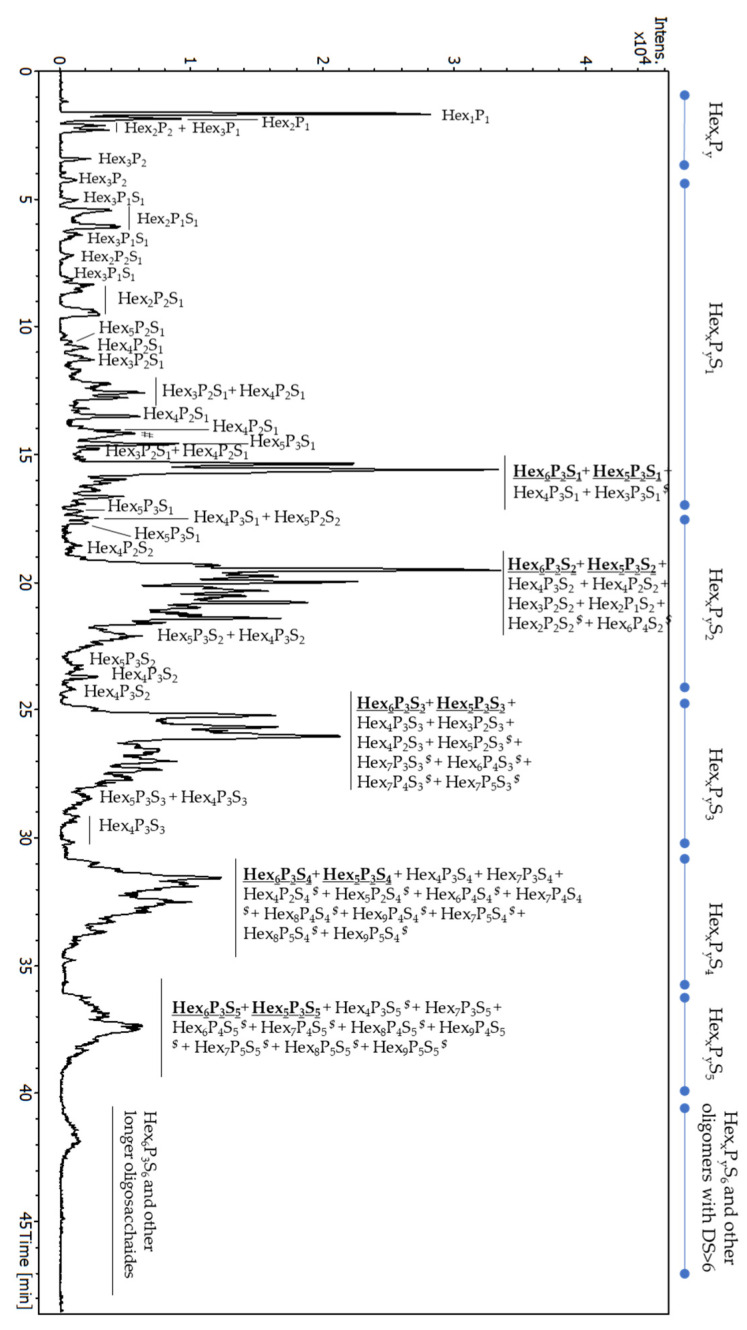
IPRP-HPLC/ESI-QTOF-MS chromatogram of S-TSP hydrolysed by cellulase and xyloglucanase. Hex—hexose (glucose or galactose, 162 Da), P—pentose, (xylose, 132 Da), S—sulphate (SO_3_-, 80 Da); the numbers in subscript indicate the number of hexoses and pentoses within the detected oligosaccharide. The most abundant oligomers Hex_6_P_3_S_x_ and Hex_5_P_3_S_x_ are underlined and in bold. $—oligomers with the intensity lower than 500 (the intensity of the highest peaks Hex_6_P_3_S_1_/Hex_5_P_3_S_1_ and Hex_6_P_3_S_2_/Hex_5_P_3_S_2_ are higher than 1 × 10^4^).

The S-TSP_1, with a DS value of two, hydrolysed with the mixture of cellulase and xyloglucanase, was analysed by ion-pair reversed phase high performance liquid chromatography (IPRP-HPLC) coupled with ESI-QTOF-MS (Figure 8). IPRP-HPLC is widely used for sulphated oligosaccharides due to its compatibility with ESI-MS and selectivity towards sulphated oligomers, including positional isomers [15,30,31]. LC-MS analysis showed well-defined groups of oligomers with different sulphate group numbers (from zero to at least six). Interestingly, only short neutral fragments were detected as minor components, suggesting that sulphation occurs in a random but relatively homogenous way along the polymer, and non-sulphated oligomers longer than Hex_3_P_2_ were not detected.

**Figure 9:**
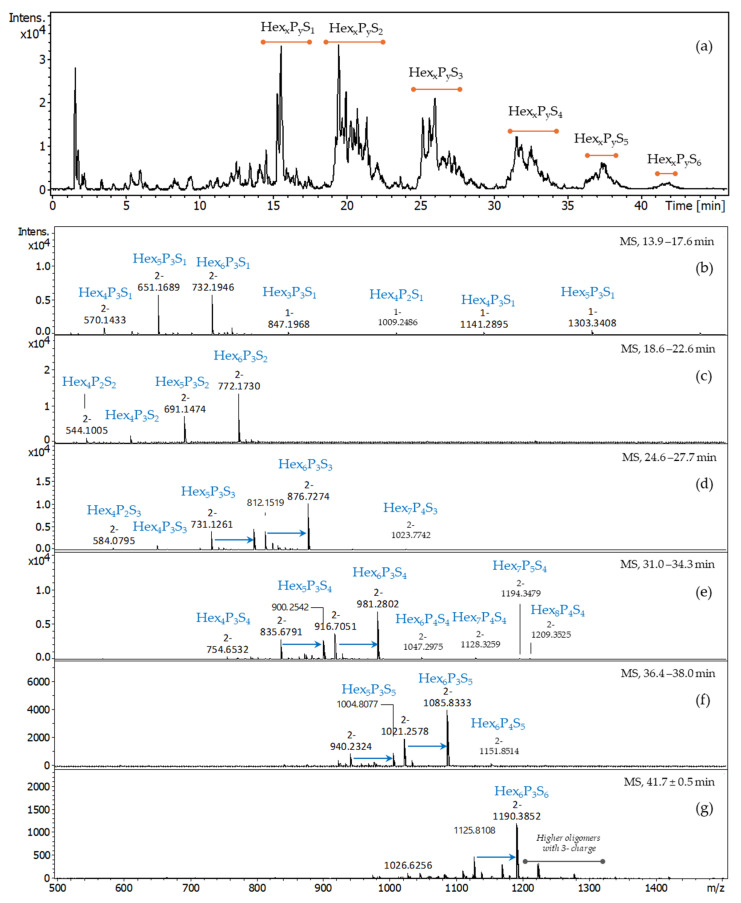
LC-MS chromatogram (**a**) and MS spectra of the most abundant peaks co-eluted in the regions of mono (**b**), bi- (**c**), tri- (**d**), tetra- (**e**), penta- (**f**) and hexasulphated (**g**) oligomers. Because various oligomers with the same sulphation degree, including positional isomers, are co-eluted, the mass spectra were averaged for their retention time range: 13.9–17.6 min ((**b**), Hex_x_P_y_S_1_); 18.6–22.6 min ((**c**), Hex_x_P_y_S_2_); 24.6–27.7 min ((**d**), Hex_x_P_y_S_3_); 31.0–34.3 min ((**e**), Hex_x_P_y_S_4_); 36.4–38.0 min ((**f**), Hex_x_P_y_S_5_); 41.7 ± 0.5 min ((**g**), Hex_x_P_y_S_6_). Hex—hexose (glucose or galactose, 162 Da), P—pentose, (xylose, 132 Da), S—sulphate (SO_3_^−^, 80 Da); DBA—dibutylamine (129 Da, adducts are indicated with blue arrow); the numbers in subscript indicate the number of hexoses and pentoses within the detected oligosaccharide. The *m*/*z* values and the corresponding ion forms are reported in Table S3.

As expected, the profile (Figure 8) suggests significantly higher structural heterogeneity of the S-TSP compared to the starting material. The increased complexity of the resulting oligosaccharide mixture is caused by non-specific sulphation reactions, leading to various positional isomers as well as enzymatic specificity of both xyloglucanase and cellulase that probably do not recognise highly sulphated regions. The extracted ion chromatograms (EICs) for *m*/*z* 732.19 (Hex_6_P_3_S_1_), 651.17 (Hex_5_P_3_S_1_), 772.18 (Hex_6_P_3_S_2_), 691.15 (Hex_5_P_3_S_2_), 876.73 (Hex_6_P_3_S_3_), 731.13 (Hex_5_P_3_S_3_), 981.28 (Hex_6_P_3_S_4_) and 900.26 (Hex_5_P_3_S_4_) are reported in Supplementary Material (Figure S6) as an example. Multiple peaks of each *m*/*z* confirm the presence of numerous positional isomers. The mass spectra of the most abundant oligomers co-eluted in the regions of mono-, di-, tri-, tetra-, penta- and hexasulphated oligosaccharides are shown in Figure 9.

Even if the ESI-MS cannot be considered quantitative because the ionisation of oligomers of different length and DS may vary, it is evident that the most abundant oligomers have the same backbone (Hex_6_P_3_S_x_ and Hex_5_P_3_S_x_) as for the digested neutral TSP, suggesting that sulphation did not significantly affect the enzymatic cleavage of the polysaccharide chain. Assuming that the enzymes would not recognize the sulphated glucose backbone, we suppose that sulphation occurred prevalently at the level of external galactose residues. These results are in agreement with the 2D NMR results (discussed below). The presence of oligomers of the same length but with the number of sulphates higher than the theoretical number of primary hydroxy-groups (CH_2_OH of non-substituted glucose and galactose) suggests that sulphation may also occur on secondary alcohol of the residues.

Additionally, the presence of longer oligomers (Figure 9) indicates that sulphation also happened on the backbone of the structure, on the glucose, in the proximity to the enzyme’s cleavage site, thus inhibiting the enzymatic cleavage.

The presence of trace oligomers such as Hex_5_P_4_S_4_ and Hex_8_P_6_S_4_ (Table S3) suggests that regions poorly substituted with galactoses are present within the TSP chains.

#### 2.3.3. NMR of Enzymatically Hydrolysed S-TSP

To confirm the LC-MS results, NMR analysis was also performed.

**Figure 10:**
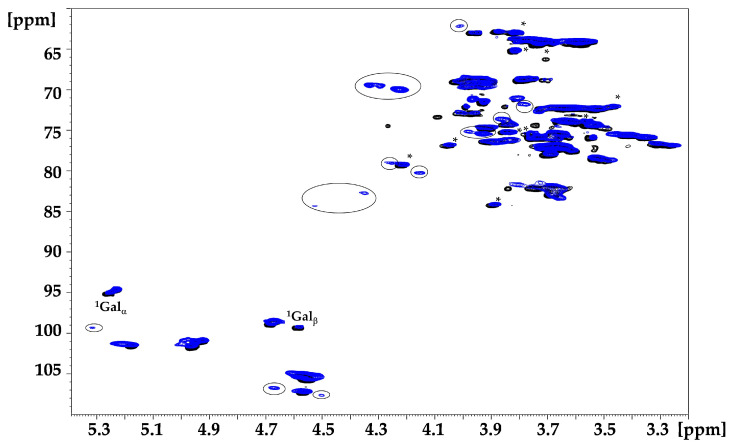
^1^H-^13^C HSQC superimposition of TSP in black hydrolysed with cellulase and S-TSP_1 hydrolysed with xyloglucanase and cellulase in blue. Circled signals are related to sulphation. Galactose anomeric signals, α and β, are reported in the figure. The superscript corresponds to the carbon number of the observed monosaccharide, which is in bold, while the monosaccharide linked is in the parentheses. * signals assigned to the cellulose enzyme; Gal—galactose.

In the HSQC spectra of the hydrolysed sulphated sample with both enzymes, new signals appeared compared to the spectrum of TSP hydrolysed with cellulase (Figure 10). The presence of CH_2_ signals at about 69 ppm for ^13^C indicated that sulphation mainly occurred on the primary alcohols of glucose and galactose residues, as previously suggested by the NMR and LC-MS results. The signals at 4.22/70.0 and 4.31/69.4 ppm were attributed respectively to the presence of the sulphated group on the position C6 of the galactose and glucose, in accordance with literature [27,31]. Moreover, the substitution on galactose led to a shift of the hydrogen in position 1 to lower fields, as shown in the literature [32,33]. Other new signals were present, arising from sequence effects following sulphation or to the presence of sulphated groups on secondary alcohols, in accordance with the LC-MS results, but extensive fractionation steps will be necessary for complete attribution.

### 2.4. Mucoadhesion

Mucoadhesion, the ability of the polymer to adhere to the mucus layer due to interactions with mucins [34,35], is an important property for the application of TSP, especially in the ophthalmic field [36]. For this reason, mucoadhesion was also studied for the sulphated products. A variety of analytical approaches can be used to evaluate the mucoadhesion properties of the polysaccharide. Here, rheology and the zeta potential were employed, based on Graça et al. [37].

#### 2.4.1. Rheology

**Figure 11:**
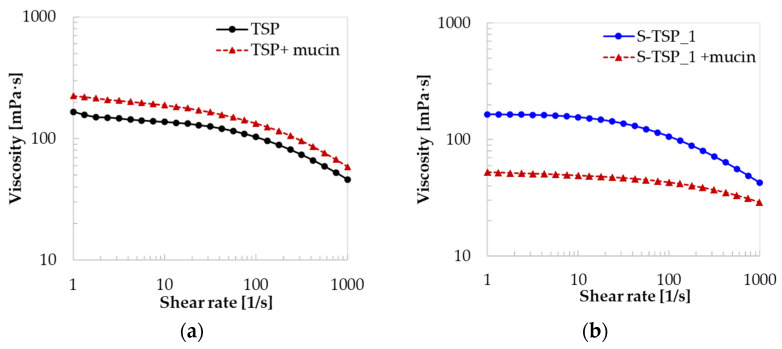
Viscosity curves: (**a**) TSP (10 mg/mL) in black and TSP with mucin (2.5 *w*/*w*) in red; (**b**) S-TSP_1 (10 mg/mL) in blue and S-TSP_1 with mucin (2.5 *w*/*w*) in red.

The viscosities of solutions of TSP and S-TSP_1 at 10 mg/mL with and without mucin (2.5 *w*/*w*) were measured, and their viscosity curves are reported in Figure 11.

Changes in viscosity were observed in the presence of mucin for TSP and S-TSP (Figure 11), demonstrating that both TSP and S-TSP interact with the glycoprotein. TSP with mucin showed a higher viscosity than the solution without the glycoprotein, but, surprisingly, the sulphated product, S-TSP, exhibited decreased viscosity in the presence of mucin. These two different behaviours reflect the fundamental structural differences between TSP and S-TSP combined with the different interactions that these result in. Sulphation of a polymer may be expected to stiffen the polymer chains as a consequence of charge repulsion, leading to decreased conformational flexibility, but the appended charged groups also carry increased volumes of associated water molecules, effectively increasing their volume. These effects cannot readily be deconvoluted, and the manner in which these charged groups influences subsequent interactions with mucin is also difficult to predict. For TSP, viscosity increased after cooperative inter/intrapolymer interactions between mucin and the polysaccharide, whereas in the case of S-TSP the interaction with the glycoprotein probably led to a decrease of the hydrodynamic volume, perhaps through tighter association, driven by charge interactions, including cation-mediated interactions, and so to decreased viscosity [38].

#### 2.4.2. Zeta Potential

The adhesion of the polysaccharides to mucin leads to changes in the surface properties such as the zeta potential (Zp) [39]. Table 4 shows the Zp of solutions of TSP and S-TSP with and without mucin.

The solution of TSP with mucin showed a similar Zp to the solution of the glycoprotein, showing that the interaction with mucin is not based on electrostatic forces, since TSP is a neutral polysaccharide [36]. Instead, the presence of mucin led to a significant change of Zp for S-TSP, which demonstrates a probable electrostatic interaction between the protein and the sulphated product. Indeed, S-TSP interacted with the positively charged domain of the mucin, leading to charge compensation and variation in the Zp, as reported in the literature for negatively charged polysaccharides [40]. We conclude that the S-TSP samples showed an ability to interact with mucin.

## 3. Discussion

In this study, synthesis and characterisation of sulphated TSP samples were achieved. The sulphated reaction was carried out successfully in organic solvent (in aqueous media it was unsuccessful) at room temperature without depolymerisation, providing varied extents of sulphation with different concentrations of reagent. The concentration of TSP and the TSP/sulphation reagent ratio are critical and can be used to control the sulphation reaction.

The success of the sulphation reaction was established using three different techniques: conductimetric titration, FT-IR spectroscopy and analysis of the zeta potential. The first method allowed the calculation of the number of sulphated groups per repeating unit of the TSP and the increase in DS with increasing concentration of reagent. Two new bands in the FT-IR spectra demonstrated the formation of new bonds consistent with sulphated groups, and the negative Zp confirmed the addition of negatively charged groups, whose presence also increased the solubility of the product due to electrostatic repulsions of the charges [41]. Moreover, the addition of sulphates bestowed new biological activities on the products, as reported above.

The physical properties of the sulphated samples were also studied to verify the maintenance of the properties of TSP, including high molecular weight and pseudoplastic behaviour. S-TSPs showed higher molecular weights than TSP, consistent with the DS obtained. The mild conditions of the reaction guarantee the maintenance of polymer chain length and the structure of TSP. The viscosity of S-TSP at low shear is very similar to TSP (Figure 4) but is maintained better at higher shear rate, indicating stronger intermolecular interactions. The presence of sulphated groups led to lower viscosity: in general, the sulphation of a polysaccharide tends to reduce the viscosity of the solution, mainly due to the electrostatic repulsion between the polymer chains, which prevents aggregation and favours a more compact conformation. Moreover, S-TSP tends to be more soluble in water than its unsulphated version: increased solubility can reduce the interaction between chains, leading to a decrease in the viscosity of the solution.

To study in detail the structure of sulphated products, an enzymatic hydrolysis step was necessary, which allows for the reduction of molecular weight without altering the chemical structure, thereby generating oligosaccharides that can also be characterised using LC-MS techniques [42]. The presence of sulphate groups inhibits the action of enzymes specific to TSP, likely due to the steric hindrance of the anionic group, which prevents, or slows down, cleavage of the β (1–4) glucose-glucose bonds. By studying the hydrolysis products of S-TSP using NMR, partial sulphation of the C6 positions of glucose and galactose was first demonstrated, as evidenced by the presence of signals in the HSQC spectrum corresponding to both the CH_2_OH and CH_2_OR groups. Additionally, it was shown that even the secondary alcohols of the monosaccharide subunits can react, since new HSQC correlation peaks appeared in the anomeric region, likely indicating a substitution at position 2 of the sugar. This finding is also supported by the HILIC/ESI-QTOF-MS chromatogram of the hydrolysed sulphated product. As expected, sulphation was not selective, and the preference for primary hydroxyl groups was confirmed. This resulted in the generation of various positional isomers that occur in a random but relatively homogeneous manner along the polymer. The complexity of the LC-MS profiles of the digested S-TSP may also be affected by the α/β-anomers. For precise determination of the sulphation position, size fractionation steps are required prior to NMR and/or LC-MS analyses.

The interaction of biomaterials with mucin potentially leads, under physiological conditions, to mucoadhesion, the ability of a polysaccharide to adhere to mucosal surfaces, such as those in the mouth, nose, intestine or genitourinary tract; these phenomena are particularly relevant to fields including pharmacology, medicine and biomedical technologies [43]. An interesting result was obtained from the study of the interaction between S-TSP and mucin, a glycoprotein involved in the mucus adhesion process. This study highlighted how the presence of negatively charged groups with significant steric hindrance along the TSP chain led to an interaction with mucin, specifically involving these charges, as evidenced by a change in Zp. This interaction differs from the one between TSP and the glycoprotein, which does not involve charges, but is likely to arise from steric interactions.

This distinct interaction produces opposing effects in terms of viscosity: in the case of TSP with mucin, there is an increase in viscosity compared to the native TSP, indicating the formation of a complex with lower structural density. Conversely, with S-TSP, the complex that forms is likely to have higher structural density.

Indeed, high molecular weight negatively charged samples are more flexible and can link distant domain of mucin, reducing the hydrodynamic volume and, consequently, the viscosity [44,45]. The decrease of viscosity with S-TSP may be advantageous, for example, in formulations for treating eye diseases, where increasing the viscosity can cause irritation to the eye [46].

In the future, analysis of the toxicity, biocompatibility and biological activities of the sulphated products will be investigated. The applications of these samples in the cosmetic, pharmaceutical or/and food fields will be also studied.

## 4. Materials and Methods

### 4.1. Materials

TSP was provided by FARMIGEA (Pisa, Italy). Sodium azide, trimethylsilyl-3-propionic acid, hydrochloric acid (≥37%), sulphur trioxide pyridine complex, *N*,*N*-dimethylformamide, cellulase from Penicillium funiculosum (6.7 units/mg), dibutylamine, amberlite^®^ IR-120 H+, sodium hydroxide, acetic acid glacial and ammonium acetate were purchased from Sigma Aldrich (Milan, Italy); NaOH 0.1 M from Merck (Kenilworth, NJ, USA); dimethylsulfoxide from Fluka Analytical (Milan, Italy); acetonitrile and methanol from Carlo Erba (Milan, Italy); and xyloglucanase (GH5) (*Paenibacillus* sp.) from Megazyme International (Wicklow, Ireland). Ethanol (96%) was purchased from Girelli Alcool (Milan, Italy), and deuterium oxide (99.9%) from Euriso-top (Saint-Aubin, France). Deionised water (conductivity less than 0.1 μS) was prepared with an osmosis inverse system (Culligan, Milan, Italy). PolyCAL-Pullulan Std-102K and PolyCAL-DextranStd-T67K were purchased from Malvern Panalytical (Malvern, UK). When not specified, the reagents were ≥98% pure.

### 4.2. Methods

#### 4.2.1. Sulphation Reaction

1.0 gof dry TSP was suspended in dry dimethylformamide (DMF, 100 mL or 200 mL), and the mixture was stirred overnight at room temperature. Then, different mole/residue ratios of polysaccharide/SO_3_-py (1:1 and 1:2) were added: S-TSP_1 was obtained adding 2.74 g of the reagent in 100 mL of DMF, S-TSP_2 using 5.48 of sulphur trioxide-pyridine complex in 100 mL and S-TSP_3 using 5.48 g in 200 mL. The resulting finely dispersed suspension was stirred vigorously for 24 h at room temperature. After dilution with water (20 mL), a homogeneous mixture was obtained and then precipitated with alcohol (EtOH/H_2_O 70% *v*/*v*) to remove DMF, pyridine and salt excess of sulphating agent and potential degradation production of the latter (Na_2_SO_4_). The S-TSP products were recovered by centrifugation. The solid obtained after centrifugation was dissolved in water (~150 mL) and precipitated with ethanol 70% v/v. This process was repeated for a third time, and lastly the precipitate was solubilised in water and dialysed in water (c.o. = 6–8 kDa).

#### 4.2.2. 1D and 2D NMR Analyses

TSP’s ^1^H and HSQC spectra were obtained with a Bruker AVANCE 600 III apparatus (Bruker, Karlsruhe, Germany) at 313 K. About 8 mg of sample was dissolved in 0.6 mL of deuterium oxide (D_2_O). The TSP sample was stirred overnight to ensure complete solubilisation before transfer to a 5 mm NMR tube for analysis.

^1^H-NMR spectra were acquired with presaturation of residual HOD, using the Bruker zgcppr pulse program, with the following parameters: number of scans 16, relaxation delay 12 s D1, time domain 32 k points and spectral width of 18 ppm, with transmitter offset 4.7 ppm.

Heteronuclear single quantum correlation (HSQC) experiments were performed using the Bruker hsqcetgpsisp2.2 pulse program, with GARP4 decoupling. The following acquisition parameters were used: 24 scans, 16 dummy scans, relaxation delay 2 s, time domain 2048 (F2) and 256 (F1), spectral width 8 ppm (F2) and 160 ppm (F1), transmitter offset 4.7 ppm (F2) and 80 ppm (F1), and number of t1 increments 320. The 1 JC–H tune value was set to 150 Hz.

S-TSP’s HSQC spectra were obtained with a Bruker AVANCE NEO 500 MHz spectrometer (Bruker, Karlsruhe, Germany) equipped with a 5 mm TCI cryoprobe at 303 K. About 8 mg of sample was dissolved in 0.6 mL of deuterium oxide (D_2_O) with 0.002% trimethylsilyl-3-propionic acid. The S-TSP samples were stirred overnight to ensure complete solubilisation before transfer to a 5 mm NMR tube for analysis.

^1^H-NMR spectra were acquired with presaturation of residual HOD, using the Bruker zgcppr pulse program, with the following parameters: number of scans 32, relaxation delay 12 s D1, time domain 32 K points and spectral width of 11 ppm, with transmitter offset 4.7 ppm.

HSQC scans were acquired using the Bruker hsqcedetgpsisp2.2 pulse program, with the following parameters: number of scans 48, dummy scans 16, relaxation delay 2 s, time domain 2048 (F2) and 256 (F1), spectral width 7.9 ppm (F2) and 80 ppm (F1) and transmitter offset 4.7 ppm (F2) and 80 ppm (F1).

The hydrolysed with cellulase TSP sample’s HSQC spectrum was obtained with a Bruker AVANCE IIIHD spectrometer operating at a proton frequency of 500 MHz (Bruker), equipped with a 5 mm BBO probe, at 303 K. About 20 mg of sample was dissolved in 0.6 mL of deuterium oxide (D_2_O) with 0.002% trimethylsilyl-3-propionic acid and transferred to a 5 mm NMR tube for analysis.

HSQC scans were acquired using the Bruker hsqcedetgpsisp2.2 pulse program, with the following parameters: number of scans 20, dummy scans 16, relaxation delay 2 s, time domain 2048 (F2) and 320 (F1), spectral width 7.9 ppm (F2) and 120 ppm (F1) and transmitter offset 4.7 ppm (F2) and 70 ppm (F1).

The hydrolysed with xyloglucanase TSP sample’s HSQC spectrum was obtained with a Bruker AVANCE IIIHD spectrometer operating at a proton frequency of 500 MHz (Bruker), equipped with a 5 mm BBO probe, at 303 K. About 20 mg of sample was dissolved in 0.6 mL of deuterium oxide (D_2_O) with 0.002% trimethylsilyl-3-propionic acid and transferred to a 5 mm NMR tube for analysis.

HSQC scans were acquired using the Bruker hsqcedetgpsisp2.2 pulse program, with the following parameters: number of scans 64, dummy scans 16, relaxation delay 1.5 s, time domain 1024 (F2) and 320 (F1), spectral width 9.9 ppm (F2) and 160 ppm (F1) and transmitter offset 4.7 ppm (F2) and 85 ppm (F1).

The hydrolysed S-TSP sample’s HSQC spectrum was obtained with a Bruker AVANCE NEO 500 MHz spectrometer (Bruker, Karlsruhe, Germany) equipped with a 5 mm TCI cryoprobe, at 303 K. About 20 mg of sample was dissolved in 0.6 mL of deuterium oxide (D_2_O) with 0.002% trimethylsilyl-3-propionic acid and transferred to a 5 mm NMR tube for analysis.

HSQC scans were acquired using the Bruker hsqcedetgpsisp2.2 pulse program, with the following parameters: number of scans 20, dummy scans 16, relaxation delay 2 s, time domain 1024 (F2) and 320 (F1), spectral width 7.9 ppm (F2) and 120 ppm (F1) and transmitter offset 4.7 ppm (F2) and 70 ppm (F1).

All spectra were processed with BrukerTopspin software version 4.1.1.

#### 4.2.3. FT-IR

The infrared spectra of TSP and S-TSP were recorded using an Alpha spectrometer (Bruker, Bremen, Germany) in the range of 4000–400 cm^−1^ at room temperature. An ATR (attenuated total reflection) (Bruker, Bremen, Germany) platinum diamond was used for the measurement. A resolution of 4 cm^−1^ and a phase resolution of 32 were employed. The sample scan time and the background scan time were both 100 scans. The data were analysed using OPUS software version 7.0 (Bruker, Bremen, Germany)

#### 4.2.4. Zeta Potential

The zeta potential was measured using a Zetasizer Nano ZS apparatus (Malvern Panalytica, Malvern, UK) with a fixed scattering angle of 173° and a 633 nm helium–neon laser. Data were analysed using Zetasizer software version 7.11 (Malvern Panalytical, UK). TSP and S-TSP were diluted in deionised water to the desired concentration (2.00 mg/L). The measurement was performed at 25 °C, five measurements, 10 run, 60 s time delay and general purpose method for the acquisition using disposable folded capillary cells (DTS1070, Malvern Panalytical, UK).

#### 4.2.5. Determining Degree of Sulphation Using Conductometric Titration

The sulphation degree of S-TSP was determined by conductimetric titration adapting the method proposed by Alekseeva et al., 2020 [29]. The analyses were performed with an automatic “Titrando” 888 (“Metrohn”, Herisau, Switzerland) titrator coupled with a Metrohm Conductimeter 712 with a conductivity cell characterised by a constant of 0.76 cm^−1^. An aqueous solution (10 mL) of each sample (120 mg), previously exchanged on an activated Amberlite^®^ IR-120(H^+^) (Sigma Aldrich, Milan, Italy) column (20 mL), was titrated by adding point by point 150 µL of 0.1 M NaOH solution every 200 s to a maximum volume of 6 mL. The number of sulphated groups on the repetitive unit was calculated using the following equation:

DS(mole) = (mmole NaOH added)/((mg S-TSP − (mmole NaOH added × 80 g/mole))/(1207 g/mole))(1)

where “1207 g/mole” is the weight of an average repeating unit of TSP and “80 g/mole” is the weight difference between the group –SO_3_H and the group –OH.

#### 4.2.6. Molecular Weight Distribution by Size Exclusion Chromatography with Triple Detector Array (HP-SEC-TDA)

Chromatographic acquisitions were performed on a Viscotek system model TDA302 (Malvern Panalytical, UK) equipped with a triple detector array exploiting simultaneous action of a refraction index detector (RI), Right and Low Angle Light Scattering (RALS and LALS) and a Viscometer (DP).

Measurements were performed at 40 °C using 2× TSKGMPWXL colums, 13 μm, 7 mm ID × 30 cm L, in series (Tosoh Bioscience, Tokyo, Japan)). AcONa 0.3 M + NaN_3_ 0.05% pH~8, prefiltered (0.22 µm mixed cellulose ester filter) was used as mobile phase at a flow rate of 0.6 mL/min. Chromatographic profiles were elaborated using OmniSEC software version 4.6.2. RI increments, referred to as *dn*/*dc*, were determined to enable conversion of RI values into concentrations of TSP and products-TSP. *dn*/*dc* values equal to 0.139 and 0.125 were calculated for TSP and S-TSP, respectively.

The detectors were calibrated with Pullulan standard, with certified molecular weight, polydispersion index and intrinsic viscosity (PolyCAL-PullulanStd-102K Malvern Panalytical, UK). Samples were analysed at 1 mg/mL in AcONa 0.3 M + NaN_3_ 0.05% pH~8.

#### 4.2.7. Rheology

The rheological properties were studied using a Modular Compact Rheometer MCR 92 (Anton Paar GmbH, Graz, Austria), with measure system DG26.7 (double gap geometry with a cylinder spindle) at a temperature of 20 °C.

Viscosity measurements were performed in rotation mode, investigated in the range of 1–1000 s^−1^, with a logarithmic ramp, and ten points per decade were acquired. Samples were solubilised in deionised water to a concentration of 10 mg/mL; about 60 mg was solubilised in 6 mL of deionized water.

#### 4.2.8. Interaction with Mucin

The interaction with mucin was evaluated comparing the viscosity of TSP and S-TSP without and with mucin. A solution of mucin 5% (*w*/*w*) was prepared by solubilising 1 g of mucin in 20 mL of deionised water.

TSP and S-TSP solutions were prepared at 10 mg/mL in deionised water, and the viscosity was measured.

For experiments with mucin, 60 mg of TSP or S-TSP were solubilised in 3 mL of deionized water. After complete solubilisation, 3 mL of mucin solution was added to obtain a final concentration of 10 mg/mL for the polysaccharide and 2.5% of mucin (*w*/*w*).

Viscosity measurements were performed in rotation mode and investigated in the range of 1–1000 s^−1^, with a logarithmic ramp, and ten points per decade were acquired.

The interaction was also studied measuring Zp. In total, 20 mg of TSP or S-TSP was solubilised in 5 mL of deionised water, and 200 mg of mucin was solubilised in 20 mL of deionised water (1% *w*/*w*). Then, 1 mL of the solution of TSP or S-TSP was added to 1 mL of mucin 1% (*w*/*w*) to a final concentration of 2 mg/mL of TSP or S-TSP and 0.5% (*w*/*w*) of mucin.

The measurement was performed at 25 °C, five measurements, 10 runs, 60 s time delay and general-purpose method for the acquisition using disposable folded capillary cells (DTS1070, Malvern Panalytical, UK).

#### 4.2.9. Enzymatic Depolymerisation

Enzymatic depolymerisations were carried out using cellulase from *Penicillium funiculosum* and xyloglucanase (GH5) from *Paenibacillus* sp. In total, 100 mg of TSP as solubilized in 18 mL of deionized water at 37 °C overnight, and 20 mg of cellulase was dissolved in 2 mL of water with low agitation overnight. After complete solubilisation, 2 mL of the cellulase solution was added to the TSP solution. A concentration ratio of TSP to enzyme of 5:1 *w*/*w* was used for hydrolysis. Then, 24 h after addition of the enzyme, the solution was heated at 100 °C for 10 min to denature the enzyme, filtered to remove the precipitated enzyme (LLG-Syringe filter, Meckenheim Germany, CA pore size 0.20 µm, Ø 13 mm) and lyophilised.

Depolymerisation of TSP with xyloglucanase was performed adapting the method used by Zhang and Ai [20]. TSP was solubilised at a concentration of 10 mg/mL in deionised water at 40 °C, dissolving 40 mg of TSP in 4 mL of deionised water overnight. Then, 20 μL of xyloglucanase (*Paenibacillus* sp., 20 U) was added to the solution. After 24 h, the reaction mixture was heated at 100 °C for 10 min to inactivate the enzyme and centrifuged at 8000 rpm for 15 min to remove insoluble materials. The supernatant was also filtered to remove the precipitated enzyme (LLG-Syringe filter, CA pore size 0.20 µm, Ø 13 mm) and then lyophilised.

For hydrolysis of S-TSP, 40 mg of S-TSP_1 was solubilised in 4 mL of deionised water at 40 °C overnight. Then, 40 μL of xyloglucanase (*Paenibacillus* sp., 20 U) and 200 μL of cellulase 10 mg/mL were added to the solution. After 24 h, the reaction mixture was heated at 100 °C for 10 min to inactivate the enzyme and centrifuged at 8000 rpm for 15 min to remove insoluble materials. The supernatant was also filtered to remove the precipitated enzyme (LLG-Syringe filter, CA pore size 0.20 µm, Ø 13 mm) and then lyophilised.

#### 4.2.10. LC/MS Analyses of Enzymatically Digested TSP and S-TSP

LC/MS analyses of both neutral and sulphated oligomers generated enzymatically were performed on an Elute UHPLC system (Bruker) coupled with an Impact II ESI-Q-TOF mass spectrometer (Bruker).

Separation of neutral TSP oligosaccharides generated by cellulase or xylogluconase was performed by hydrophilic interaction chromatography on a XBridge BEH Amide column (100 × 2.1 mm, 2.5 μm, Waters, Milford, MA, USA) at 40 °C. The eluents A (5 mM ammonium acetate in water) and B (acetonitrile) were delivered at a 0.2 mL/min flow rate. Separation was achieved by applying the following gradient: the solvent composition was held at 20% A for the first 2 min, then increased to 80% A over 23 min, where it was held at 80% A for 5 min and then returned to 20% A over 1 min, where it was held for the last 10 min for equilibrating the chromatographic column before injection of the next sample. The samples were injected at 0.1 mg/mL in 50% acetonitrile. The injected volume was 5 μL.

The sulphated S-TSP oligosaccharides generated by cellulase and xylogluconase were analysed in a ion pair reversed phase (IPRP) HPLC on a C18 Kinetex column (100 × 2.1 mm, 2.6 μm, Phenomenex, Torrance, CA, USA) using dibutylamine (DBA) acetate as the ion pair reagent. Eluent A (10 mM DBA, 10 mM acetic acid in water) and eluent B (10 mM DBA and 10 mM acetic acid in methanol) were delivered at 0.15 mL/min. The separation of oligomers with different lengths and sulphation degrees was performed keeping the column at 35 °C and using the following gradient: the solvent composition was held at 7% B for the first 5 min, then increased to 45% B over 35 min, and up to 90% B over another 10 min, where it was held at for 7 min; afterwards, it was returned to 7% B over 3 min, and the column was equilibrated for the last 30 min. Samples were analysed at a concentration of 1 mg/mL, and the injection volume was 2 μL.

The mass spectra were acquired in negative ion mode (capillary voltage 4 kV) in the *m*/*z* 140–2500 mass range. Nitrogen was used as a drying (7 L/min) and nebulizing (1.8 bar) gas, and the ion transfer capillary was kept at 200 °C.

## 5. Conclusions

In conclusion, a method for controlled sulphation of the TSP polymer that avoids depolymerisation has been developed, and its structural and physico-chemical properties have been characterised with analytical approaches, such as NMR, LC/MS and HP-SEC-TDA.

Unexpectedly, the viscosity of a mixture of S-TSP and mucin, serving as a measurement of mucoadhesion, was lower than either intact TSP or TSP with mucin, highlighting the difficulty of predicting the outcome of polymer interactions but providing a route by which these properties may be controlled and, hence, by which the applications of TSP-based polymers can be influenced.

## Figures and Tables

**Table 1:** Reaction conditions for sulphated TSP, S-TSP, synthesis (molar ratio and concentration), resulting in sulphation degree and zeta potential.

Sample	Temperature (°C)	TSP/SO_3_xPy Molar Ratio	TSP Concentration in DMF (mg/mL)	Sulphation Degree (DS) *	Zeta Potential (mV)
TSP	-	-	-	-	−0.70 ± 0.16
S-TSP_1	RT	1:1	10	2	−43.0 ± 1.02
S-TSP_2	RT	1:2	10	3	−47.4 ± 2.41
S-TSP_3	RT	1:2	5	4	−58.1 ± 3.12

* by conductimetric titration; RT: room temperature.

**Table 2:** HP-SEC-TDA results for pristine and modified TSP: Mw—weight average molecular weight, Mn—number average molecular weight, Pd—polydispersity, [η]—intrinsic viscosity, Rh—hydrodynamic radius, *a*—Mark–Houwink parameter.

Sample	Mw (kDa)	Mn (kDa)	Pd (Mw/Mn)	[η] (dL/g)	Rh (nm)	*a*
TSP	620	405	1.5	5.1	35	0.78
S-TSP_1	750	485	1.5	3.8	34	0.82
S-TSP_2	810	540	1.5	4.1	36	0.80
S-TSP_3	1000	730	1.4	4.5	41	0.81

**Table 3:** Attribution of the HSQC’s signal of hydrolysed TSP. The observed monosaccharide is in bold, while the monosaccharide linked is in the parentheses. Glc-glucose; Xyl-xylose; Gal-galactose.

Monosaccharide Residues	Chemical Shift (ppm) H/C					
	1	2	3	4	5	6
**Glc_NR_**	4.55/105.2	3.33/75.7	3.51/78.2	3.51/72.1	3.69/77.0	3.77/68.5
**Glc**-(Xyl)	4.55/105.2	3.40/75.4	3.67/76.8	3.67/81.9	3.81/76.0	3.99–3.92/68.7
**Glc**-(Xyl-Gal)	4.55/105.2	3.40/75.4	3.67/76.8	3.67/82.6	3.89/76.1	3.95–3.91/69.4
**Glc_α_**	5.21/94.4	3.62/73.8	3.82/74.1	3.68/81.9	3.93/72.6	3.86/62.6
**Glc_β_**	4.65/98.3	3.29/76.5	3.65/77.1	3.68/81.7	3.60/77.2	3.93–3.81/62.7
**Xyl**-(Gal)	5.16/101.4	3.66/82.7	3.90/74.6	3.71/72.1	3.71–3.56/63.9	-
**Xyl**-(Glc)	4.95/101.6	3.54/74.1	3.72/75.7	3.59/72.1	3.71–3.56/63.9	-
**Xyl**-(Glc_NR_)	4.93/100.9	3.54/74.1	3.72/75.7	3.63/72.1	3.71–3.56/63.9	-
**Gal**	4.55/107.2	3.60/73.8	3.65/75.1	3.91/71.3	3.67/77.7	3.77/63.7

**Table 4:** Zeta potential of solutions of mucin 0.5 *w*/*w*, TSP and S-TSP_1 at 2 mg/mL without and with mucin.

Sample	Zeta Potential (mV)
Mucin	−2.59 ± 0.13
TSP	−0.70 ± 0.16
TSP + mucin	−2.09 ± 0.15
S-TSP_1	−43.0 ± 1.02
S-TSP_1 + mucin	−10.1 ± 0.75

## Data Availability

The original contributions presented in the study are included in the article and Supplementary Materials, further inquiries can be directed to the corresponding author.

## Supplementary Materials

The following supporting information can be downloaded at: https://www.mdpi.com/article/10.3390/molecules29235510/s1, Figure S1: Chromatographic profile (red—refractive index; black—low laser light scattering; green—right angle light scattering; blue—viscometer) of S-TSP_2 (a) and S-TSP_3 (b).; Figure S2: ^1^H spectra of TSP in black, S-TSP_1 in dark blue, S-TSP_2 in blue and S-TSP_3 in light blue. The peaks of the anomeric protons of the residues and position 2 of glucose are indicated. The superscript corresponds to the carbon number of the observed monosaccharide, labelled in bold, while the monosaccharide linked is shown in parentheses. Glc-glucose; Xyl-xylose; Gal-galactose.; Figure S3: ^1^H-^13^C HSQC superimposition of TSP in black with partial assignments and S-TSP_1 in blue. The superscript corresponds to the carbon number of the observed monosaccharide, which is in bold, while the monosaccharide linked is shown in parentheses. Signals related to sulphation are circled in blue. Arabinose signals are circled in grey. Glc-glucose; Xyl-xylose; Gal-galactose.; Figure S4: ^1^H-^13^C HSQC anomeric region of hydrolysed TSP with cellulase. Signals related to galactose anomeric signals, α and β. The superscript corresponds to the carbon number of the observed monosaccharide, which is in bold, while the monosaccharide linked is shown in parentheses. Glc-glucose; Glc_red_-glucose reducing end; Xyl-xylose; Gal-galactose.; Figure S5: integration of ^1^H-^13^C HSQC anomeric region of hydrolysed TSP with xyloglucanase in blue. The superscript corresponds to the carbon number of the observed monosaccharide, which is in bold, while the monosaccharide linked is shown in parentheses. Glc-glucose; Glc_red_-glucose reducing end; Xyl-xylose; Gal-galactose; Figure S6: IPRP-HPLC/ESI-QTOF-MS chromatogram of S-TSP hydrolysed by cellulase and xyloglucanase (a) and extracted ion chromatograms (EICs) showing the separation of various positional isomers of the most abundant mono-, di-, tri- and tetrasulphated Hex6P3Sx and Hex5P3Sx (b–i). Hex—hexose (glucose or galactose, 162 Da), P—pentose, (xylose, 132 Da), S—sulphate (SO_3_^−^, 80 Da); DBA—dibutylamine (129 Da); the numbers in subscript indicate the number of hexoses and pentoses within the detected oligosaccharide; Table S1: Reaction conditions for sulphated S-TSP_1a, synthesis (molar ratio and concentration) and resulting sulphation degree and zeta potential; Table S2: HP-SEC-TDA results: Mw—weight average molecular weight, Mn—number average molecular weight, Pd—polydispersity, µ—intrinsic viscosity, Rh—hydrodynamic radius, a—Mark–Houwink parameter; Table S3: MS data of oligosaccharides identified in the S-TSP_1 digested with cellulase and xyloglucanase.
