# Supplementary material for: Synthesis and Physiochemical Properties of Sulphated Tamarind (Tamarindus indica L.) Seed Polysaccharide
Source: Molecules. 2024 Nov 21;29(23):5510. doi: 10.3390/molecules29235510 (PMC11643769; doi:10.3390/molecules29235510)
Supplement: Supplementary file 1 [file molecules-29-05510-s001.zip › molecules-3285613-supplementary.pdf]

Supplementary materials

Table S1. Reaction conditions for the sulphated S-TSP_1a, synthesis (molar ratio and concentration) and resulting sulphation degree and zeta potential.

Sample	Temperature (°C)	TSP/SO_3xPy molar ratio	TSP concentration in DMF (mg/mL)	Sulphation Degree (DS)*	Zeta potential (mV)**
S-TSP_1a	50	1:1	10	2.6	-37.8±3.12

*by conductimetric titration

** measurement at 40°C and at 1 mg/mL of the sample in deionized water

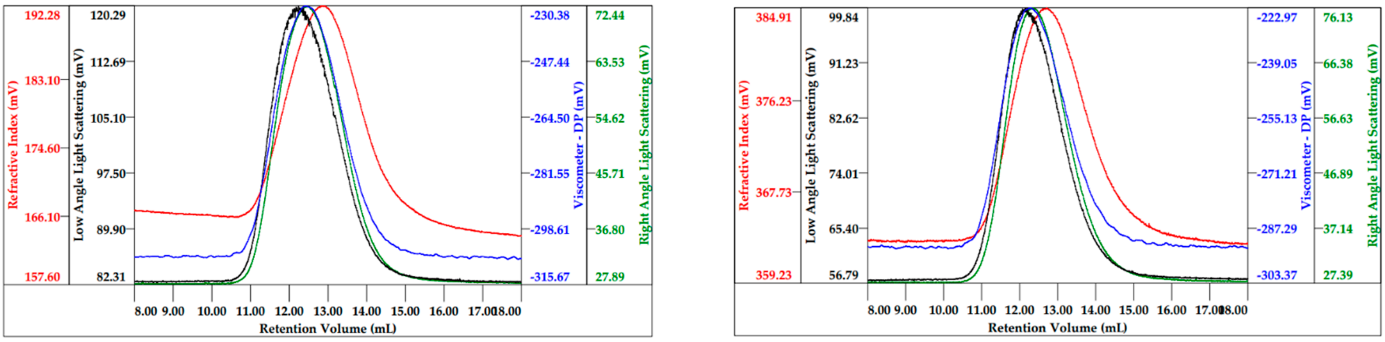

(**a**) (**b**)

Figure S1. Chromatographic profile (red—refractive index; black—low laser light scattering; green—right angle light scattering; blue—viscometer) of pristine S-TSP_2 (a) and S-TSP_3 (b).

Table S2. HP-SEC-TDA results: Mw – weight average molecular weight, Mn – number average molecular weight, Pd – polydispersity, μ – intrinsic viscosity, Rh – hydrodynamic radius, a – Mark-Houwink parameter.

Sample	Mw (kDa)	Mn (kDa)	Pd (Mw/Mn)	[η](dl/g)	Rh (nm)	*a*
S-TSP_1a	660	355	1.8	4.03	33	0.70

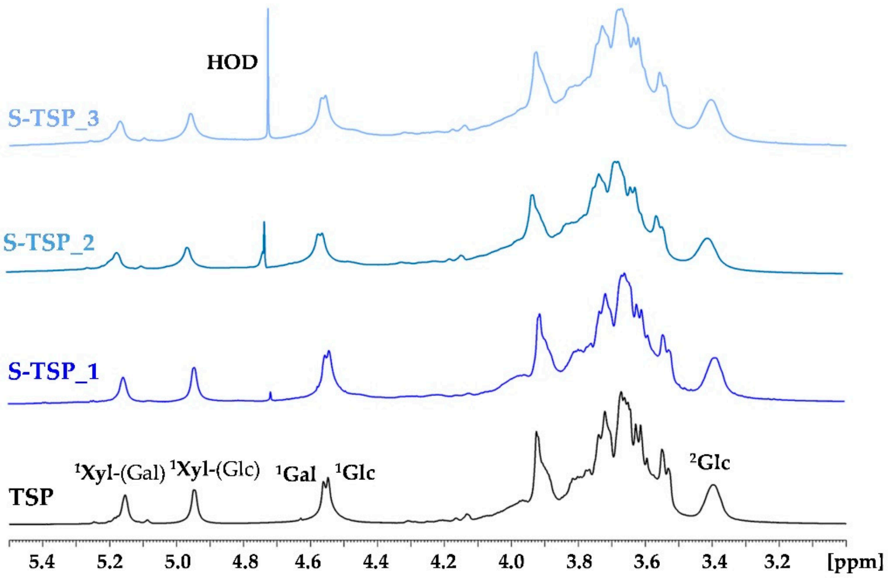

Figure S2. ^{1}H spectra of TSP in black, S-TSP_1 in dark blue, S-TSP_2 in blue and S-TSP_3 in light blue.
The peaks of the anomeric protons of the residues and the position 2 of glucose are indicated. The superscript corresponds to the carbon number of the observed monosaccharide, labelled in bold, while the monosaccharide linked is in the parentheses. Glc-glucose; Xyl-xylose; Gal-galactose.

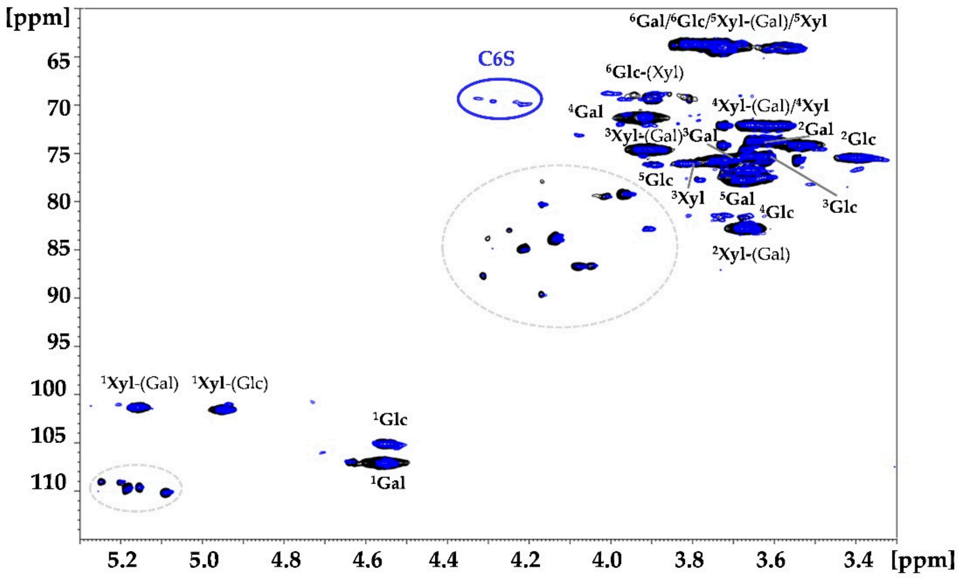

Figure S3. ^{1}H-^{13}C HSQC superimposition of TSP in black with partial assignments and S-TSP_1 in blue. The superscript corresponds to the carbon number of the observed monosaccharide, which is in bold, while the monosaccharide linked is in the parentheses. Signals related to the sulphation were circled in blue. Arabinose signals are circled in grey. Glc-glucose; Xyl-xylose; Gal-galactose.

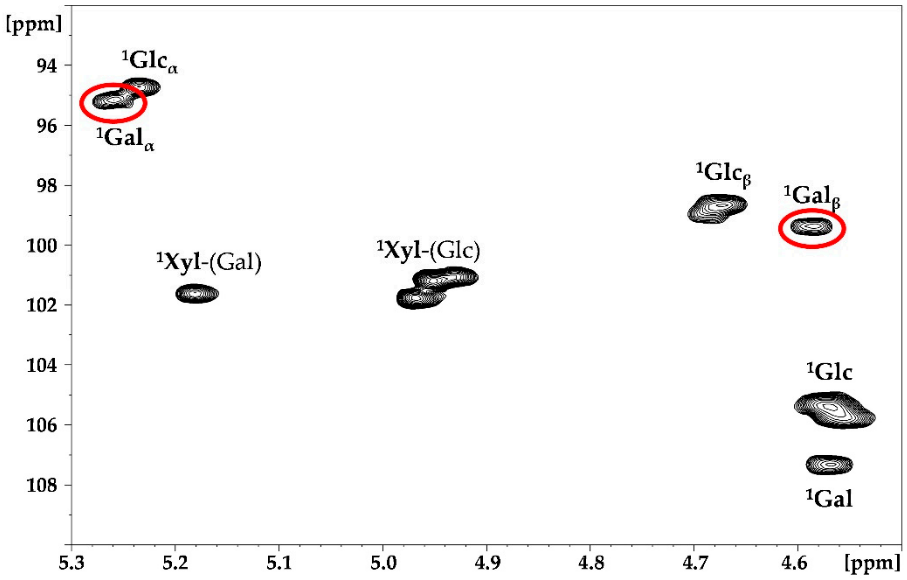

Figure S4. ^{1}H-^{13}C HSQC anomeric region of hydrolysed TSP with cellulase. Signals related to Galactose anomeric signals, a and b. The superscript corresponds to the carbon number of the observed monosaccharide, which is in bold, while the monosaccharide linked is in the parentheses. Glc-glucose; Glc_{red}-glucose reducing end; Xyl-xylose; Gal-galactose.

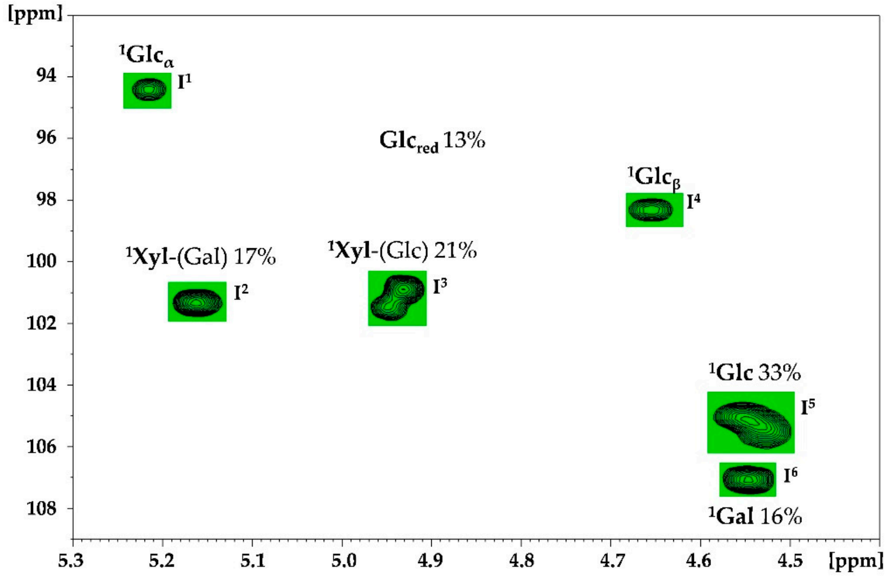

Figure S5. Integration of ^{1}H-^{13}C HSQC anomeric region of hydrolysed TSP with xyloglucanase in black. The superscript corresponds to the carbon number of the observed monosaccharide, which is in bold, while the monosaccharide linked is in the parentheses. The relative molar percentages of the individual monosaccharides are reported.

Glc-glucose; Glc_{red}-glucose reducing end; Xyl-xylose; Gal-galactose

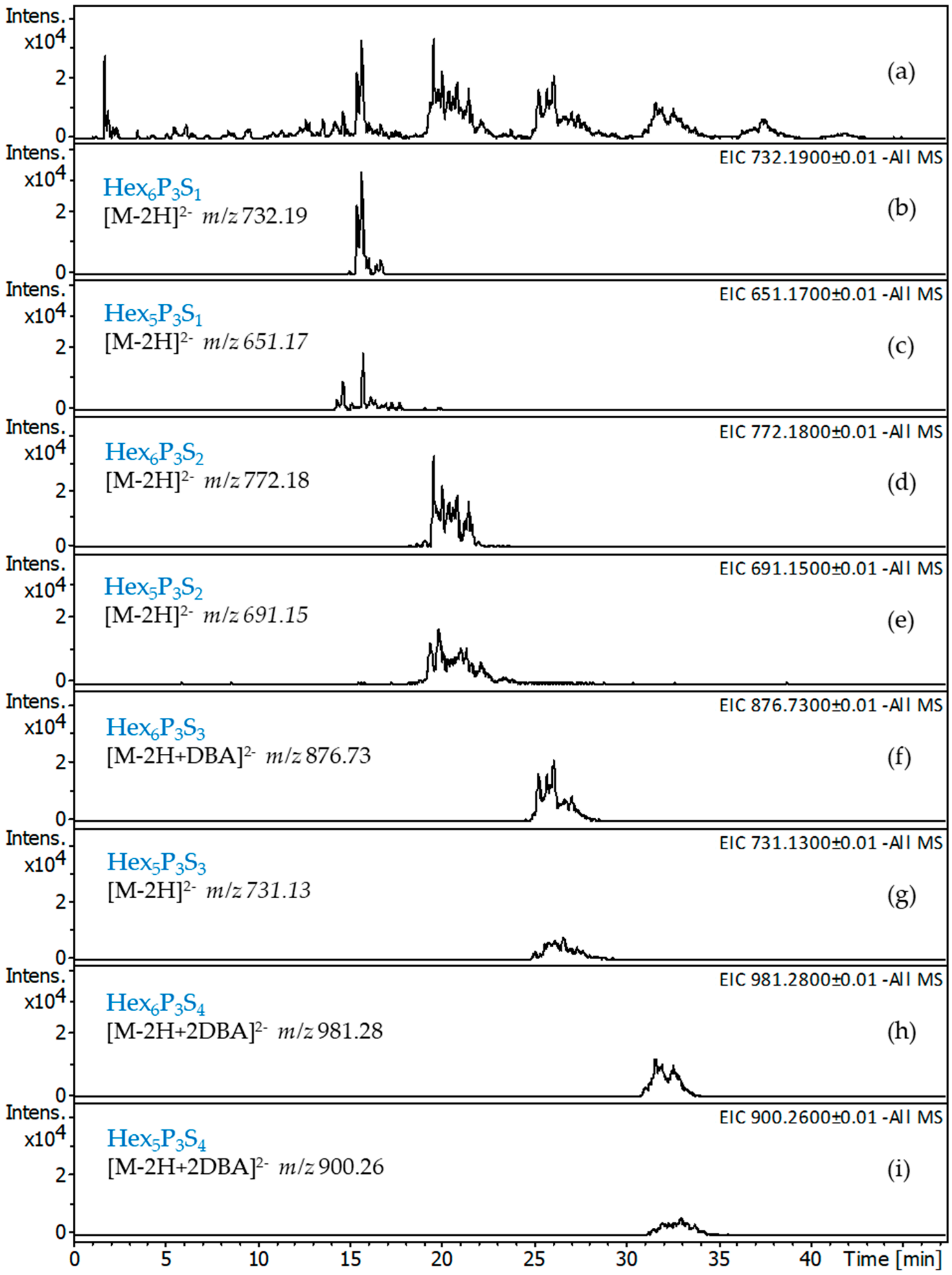

Figure S6. IPRP-HPLC/ESI-QTOF-MS chromatogram of S-TSP hydrolysed by cellulase and xyloglucanase (**a**) and extracted ion chromatograms (EICs) showing the separation of various positional isomers of the most abundant mono-, di-, tri- and tetrasulphated $Hex_6P_3S_x$ and $Hex_5P_3S_x$ (**b-i**).

Hex - hexose (glucose or galactose, 162 Da), P – pentose, (xylose, 132 Da), S – sulphate (SO_3^-, 80 Da); DBA – dibutylamine (129 Da); the numbers in subscript indicate the number of hexoses and pentoses within the detected oligosaccharide

Table S3. MS data of oligosaccharides identified in the S-TSP_1 digested with cellulase and xyloglucanase

	Oligosaccharide	*Monoisotopic ions observed in ESI-MS*			
		Ion form	*m/z* experimental	*m/z* theoretical	Error, ppm
No sulphated oligomers	Hex$_1$P$_1$	[M-H]$^-$	311,0971	311,0984	-4,2
		[2M-H]$^-$	623,2009	623,2040	-5,0
	Hex$_2$P$_1$	[M-H]$^-$	473,1497	473,1512	-3,2
	Hex$_2$P$_2$	[M-H]$^-$	605,1901	605,1935	-5,6
	Hex$_3$P$_1$	[M-H]$^-$	635,1984	635,2040	-8,8
	Hex$_3$P$_2$	[M-H]$^-$	767,2415	767,2463	-6,3
Mono-sulphated oligomers	Hex$_2$P$_1$S$_1$	[M-H]$^-$	553,1042	553,1080	-6,9
	Hex$_3$P$_1$S$_1$	[M-H]$^-$	715,1568	715,1608	-5,6
	Hex$_2$P$_2$S$_1$	[M-H]$^-$	685,1459	685,1503	-6,4
	Hex$_3$P$_2$S$_1$	[M-H]$^-$	847,1954	847,2031	-9,1
	Hex$_4$P$_2$S$_1$	[M-H]$^-$	1009,2486	1009,2559	-7,2
	Hex$_5$P$_2$S$_1$	[M-2H]$^{2-}$	585,1488	585,1507	-3,2
	Hex$_3$P$_3$S$_1$$^{\$}$	[M-2H]$^{2-}$	489,1173	489,1190	*-3,5*
	Hex$_4$P$_3$S$_1$	[M-2H]$^{2-}$	570,1446	570,1454	-1,4
	**Hex$_5$P$_3$S$_1$**	[M-2H]$^{2-}$	651,1684	651,1719	-5,4
	**Hex$_6$P$_3$S$_1$**	[M-2H]$^{2-}$	732,1945	732,1982	-5,1
Di-sulphated oligomers	Hex$_2$P$_1$S$_2$ $^{\$}$	[M+DBA-H]$^{1-}$	762,2108	762,2166	-7,6
	Hex$_2$P$_2$S$_2$ $^{\$}$	[M+DBA-H]$^{1-}$	894,2524	894,2513	1,2
	Hex$_3$P$_2$S$_2$	[M+DBA-H]$^{1-}$	1056,3050	1056,3117	-6,3
	Hex$_4$P$_2$S$_2$	[M-2H]$^{2-}$	544,1005	544,1027	-4,0
	Hex$_5$P$_2$S$_2$	[M-2H]$^{2-}$	625,1273	625,1291	-2,9
	Hex$_4$P$_3$S$_2$	[M-2H]$^{2-}$	610,1217	610,1239	-3,6
	**Hex$_5$P$_3$S$_2$**	[M-2H]$^{2-}$	691,1475	691,1503	-4,1
	**Hex$_6$P$_3$S$_2$**	[M-2H]$^{2-}$	772,1729	772,1766	-4,8
	Hex$_6$P$_4$S$_2$ $^{\$}$	[M-2H]$^{2-}$	838,1935	838,1978	-5,1
Tri-sulphated oligomers	Hex$_3$P$_2$S$_3$ $^{\$}$	[M-2H]$^{2-}$	503,0543	503,0547	-0,8
	Hex$_4$P$_2$S$_3$	[M-2H]$^{2-}$	584,0795	584,0811	-2,7
	Hex$_5$P$_2$S$_3$ $^{\$}$	[M-2H]$^{2-}$	665,1053	665,1075	-3,3
	Hex$_4$P$_3$S$_3$	[M-2H]$^{2-}$	650,0981	650,1023	-6,5
	**Hex$_5$P$_3$S$_3$**	[M-2H]$^{2-}$	731,1249	731,1287	-5,2
		[M+DBA-2H]$^{2-}$	795,7010	795,7046	-4,5
	**Hex$_6$P$_3$S$_3$**	[M-2H]$^{2-}$	812,1518	812,1551	-4,1
		[M+DBA-2H]$^{2-}$	876,7303	876,7310	-0,8
	Hex$_7$P$_3$S$_3$ $^{\$}$	[M+DBA-2H]$^{2-}$	957,7494	957,7574	-8,4

	$Hex_6P_4S_3$ $	$[M+DBA-2H]^{2-}$	942,7545	942,7521	-2,5
	$Hex_7P_4S_3$ $	$[M+DBA-2H]^{2-}$	1023,7742	1023,7785	-4,2
	$Hex_7P_5S_3$ $	$[M+DBA-2H]^{2-}$	1089,7941	1089,7996	-5,0
Tetra-sulphated oligomers	$Hex_4P_2S_4$ $	$[M+DBA-2H]^{2-}$	688,6337	688,6354	-2,5
	$Hex_5P_2S_4$ $	$[M+DBA-2H]^{2-}$	769,6561	769,6618	-7,6
	$Hex_4P_3S_4$	$[M+DBA-2H]^{2-}$	754,6548	754,6565	-2,3
	**$Hex_5P_3S_4$**	$[M+DBA-2H]^{2-}$	835,6804	835,6830	-3,1
		$[M+2DBA-2H]^{2-}$	900,2555	900,2588	-3,7
	**$Hex_6P_3S_4$**	$[M+2DBA-2H]^{2-}$	981,2802	981,2852	-5,1
	$Hex_7P_3S_4$	$[M+2DBA-2H]^{2-}$	1062,3061	1062,3116	-5,2
	$Hex_6P_4S_4$ $	$[M+2DBA-2H]^{2-}$	1047,2975	1047,3064	-8,5
	$Hex_7P_4S_4$ $	$[M+2DBA-2H]^{2-}$	1128,3259	1128,3328	-6,1
	$Hex_8P_4S_4$ $	$[M+2DBA-2H]^{2-}$	1209,3525	1209,3592	-5,5
	$Hex_9P_4S_4$ $	$[M+2DBA-2H]^{2-}$	1290,3777	1290,3856	-6,1
	$Hex_7P_5S_4$ $	$[M+2DBA-2H]^{2-}$	1194,3382	1194,3539	-13,1
	$Hex_8P_5S_4$ $	$[M+2DBA-2H]^{2-}$	1275,3743	1275,3803	-4,7
	$Hex_9P_5S_4$ $	$[M+2DBA-2H]^{2-}$	1356,4063	1356,4067	-0,3
Penta-sulphated oligomers	$Hex_4P_3S_5$	$[M+2DBA-2H]^{2-}$	859,2096	859,2108	-1,4
	**$Hex_5P_3S_5$**	$[M+2DBA-2H]^{2-}$	940,2331	940,2372	-4,4
		$[M+3DBA-2H]^{2-}$	1004,8057	1004,8131	-7,4
	**$Hex_6P_3S_5$**	$[M+2DBA-2H]^{2-}$	1021,2579	1021,2637	-5,7
		$[M-2H+3DBA]^{2-}$	1085,8336	1085,8395	-5,4
	$Hex_7P_3S_5$ $	$[M-2H+3DBA]^{2-}$	1166,8599	1166,8659	-5,1
	$Hex_6P_4S_5$ $	$[M+3DBA-2H]^{2-}$	1151,8572	1151,8607	-3,0
	$Hex_7P_4S_5$ $	$[M+3DBA-2H]^{2-}$	1232,8802	1232,8871	-5,6
	$Hex_8P_4S_5$ $	$[M+3DBA-2H]^{2-}$	1313,9039	1313,9135	-7,3
	$Hex_9P_4S_5$ $	$[M+3DBA-2H]^{2-}$	1394,9255	1394,9399	-10,3
	$Hex_7P_5S_5$$	$[M+3DBA-2H]^{2-}$	1298,9013	1298,9082	-5,3
	$Hex_8P_5S_5$ $	$[M+3DBA-2H]^{2-}$	1379,9202	1379,9346	-10,5
	$Hex_9P_5S_5$ $	$[M+3DBA-2H]^{2-}$	1460,9437	1460,9610	-11,8

The most abundant oligomers $Hex_6P_3S_x$ *and* $Hex_5P_3S_x$ *are in bold.*

***$ -** oligomers with the intensity lower than 500 (the intensity of the highest peaks* $Hex_6P_3S_1$/ $Hex_5P_3S_1$ *and* $Hex_6P_3S_2$/ $Hex_5P_3S_2$ *are higher than* $1*10^4$*)*
